# Protective Effect of Fucoxanthin on Zearalenone-Induced Hepatic Damage through Nrf2 Mediated by PI3K/AKT Signaling

**DOI:** 10.3390/md21070391

**Published:** 2023-07-03

**Authors:** Rebai Ben Ammar, Hamad Abu Zahra, Abdulmalek Mohammad Abu Zahra, Manal Alfwuaires, Sarah Abdulaziz Alamer, Ashraf M. Metwally, Thnaian A. Althnaian, Saeed Y. Al-Ramadan

**Affiliations:** 1 Department of Biological Sciences, College of Science, King Faisal University, Al-Ahsa 31982, Saudi Arabia; habuzahra@kfu.edu.sa (H.A.Z.); malfwuaires@kfu.edu.sa (M.A.); salamer@kfu.edu.sa (S.A.A.); ametwally@kfu.edu.sa (A.M.M.); 2 Center of Biotechnology of Borj-Cedria, Laboratory of Aromatic and Medicinal Plants, Technopole of Borj-Cedria, Hammam-Lif 2050, Tunisia; 3 Medical Laboratory Sciences Department, Jordan University of Science and Technology, Irbid 22110, Jordan; 4 Botany and Microbiology Department, Faculty of Science, Assiut University, Assiut 71516, Egypt; 5 Department of Anatomy, College of Veterinary Medicine, King Faisal University, Al-Ahsa 31982, Saudi Arabia; talthnaian@kfu.edu.sa

**Keywords:** marine algae, fucoxanthin, oxidative stress, HepG2, Nrf2, PI3K/AKT

## Abstract

Hepatotoxic contaminants such as zearalenone (ZEA) are widely present in foods. Marine algae have a wide range of potential applications in pharmaceuticals, cosmetics, and food products. Research is ongoing to develop treatments and products based on the compounds found in algae. Fucoxanthin (FXN) is a brown-algae-derived dietary compound that is reported to prevent hepatotoxicity caused by ZEA. This compound has multiple biological functions, including anti-diabetic, anti-obesity, anti-microbial, and anti-cancer properties. Furthermore, FXN is a powerful antioxidant. In this study, we examined the effects of FXN on ZEA-induced stress and inflammation in HepG2 cells. MTT assays, ROS generation assays, Western blots, and apoptosis analysis were used to evaluate the effects of FXN on ZEA-induced HepG2 cell inflammation. Pre-incubation with FXN reduced the cytotoxicity of ZEA toward HepG2 cells. FXN inhibited the ZEA-induced production of pro-inflammatory cytokines, including IL-1 β, IL-6, and TNF-α. Moreover, FXN increased HO-1 expression in HepG2 by activating the PI3K/AKT/NRF2 signaling pathway. In conclusion, FXN inhibits ZEA-induced inflammation and oxidative stress in hepatocytes by targeting Nrf2 via activating PI3K/AKT signaling.

## 1. Introduction

Zearalenone (ZEA) can beproduced by several fungi including *Fusarium graminearum* (*Gibberella zeae*), *Fusarium cerealis*, and *Fusarium culmorum*. Cereal grains and foodstuffs are commonly contaminated with ZEA [1,2]. A target organ is reached by ZEA entering the body through the hepatic and systemic vessels. A major function of the liver is to detoxify foreign substances and eliminate metabolites. ZEA is a potent hepatotoxic toxin, which may result in hepatitis, cirrhosis, and liver immunological damage [3,4]. There are many chronic liver diseases associated with ROS. Cirrhosis of the liver is characterized by an imbalance between oxidation and oxidation resistance. ZEA induces the inactivation of PI3K/AKT signaling, subsequently increasing ROS production [5]. 

The PI3K/Akt pathway has been shown to contribute significantly to cellular defense against inflammatory stimuli. The PI3K/Akt signaling pathway has been demonstrated to be involved in this process [6]. Thus, the regulation of PI3K/Akt signaling pathways may be an effective strategy to prevent ROS-induced hepatic damage. It has been demonstrated by Fan et al. that ZEA activates the secretion of TNF-α, IL-1β, IL-6, and other pro-inflammatory cytokines in the intestinal porcine enterocytes isolated from the jejunum of neonatal unsuckled piglet (IPEC-J2)cells as well as macrophages [7]. Accordingly, natural anti-inflammatory agents that are safe and effective are needed to prevent or alleviate the effects of ZEA on the liver.

In contrast, previous reports show that natural compounds inhibit hepatic injury [8,9,10] and carotenoids are abundant antioxidants present in many plants and algae [8,9,10]. Brown seaweed contains a specific carotenoid called fucoxanthin (FXN). Approximately 10% of the estimated natural production of carotenoids is accounted for by these carotenoids. The FXN-containing seaweeds are often used as a source of food in Southeast Asian countries [11,12,13,14]. In various cell culture models and animal studies, FXN has been shown to be a powerful antioxidant [15,16,17,18,19]. 

FXN and its metabolites have a high level of safety, significant bioactivities, and pharmacological properties, making them promising nutritional and medicinal ingredients [20]. FXN has many beneficial effects, including inhibiting cancer cell proliferation, promoting weight loss, acting as an antioxidant and anti-inflammatory agent, protecting intestinal health, preventing organ fibrosis, and interacting with intestinal flora [11,21,22,23]. Furthermore, FXN improved ventricular rhythm and muscle function in aging mice and improved glycolipid metabolism in type 2 diabetic mice [23]. 

FXN pretreatment can inhibit the inflammatory pain caused by ultraviolet B (UVB) as well as the exfoliation of the corneal epithelium [24]. Moreover, due to the fact that it is an edible carotenoid, FXN has a good safety profile. FXN is considered a very safe antioxidant even when it is consumed at a level of 200 mg/kg per day [25]. According to Rajendran and Zharani, pretreatment of HUVEC cells with FXN inhibited the release of pro-inflammatory cytokines by oxidizing low-density lipoprotein (Ox-LDL) [26]. Despite this, no studies have been conducted on the role of FXN in ZEA-induced liver injury. Therefore, the purpose of this study was to determine the ability of FXN to reduce the damage caused by ZEA to HepG2 cells.

## 2. Results

### 2.1. HepG2 Cells Are Protected from Hepatotoxicity Induced by ZEA by FXN

**Figure 1:**
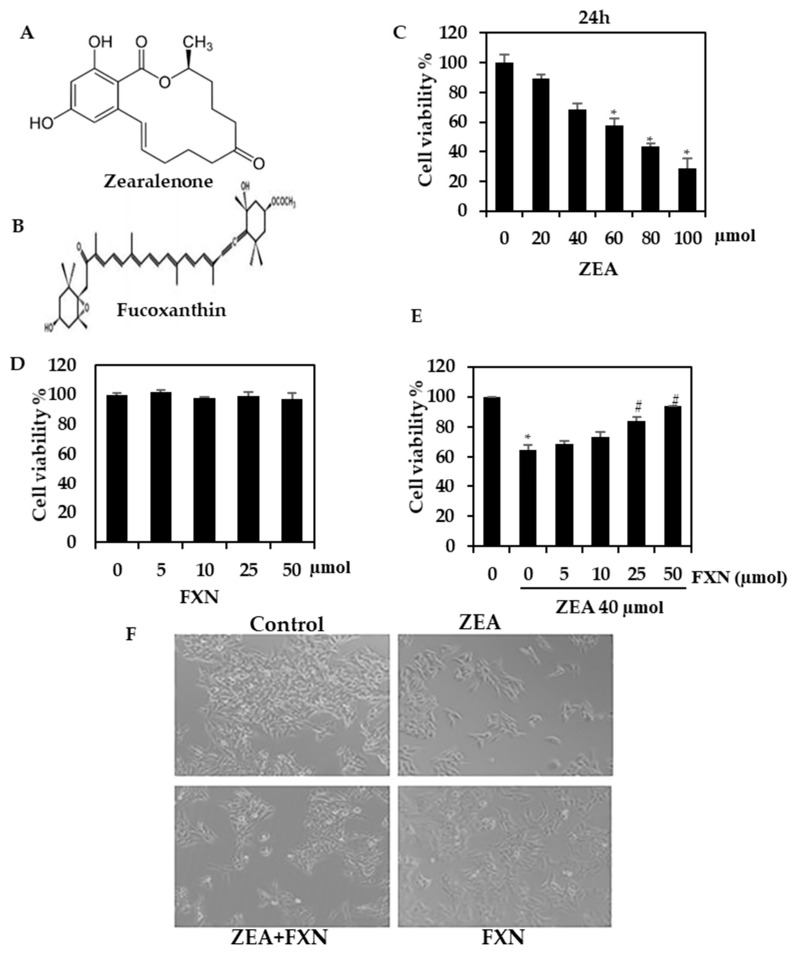
Analysis of ZEA and FXN viability. (**A**,**B**) Chemical structure of ZEA and FXN. (**C**) The viability of HepG2 cells was determined using the MTT assay after the addition of the indicated ZEA dosages for 24 h. (**D**) FXN cytotoxicity to HepG2 cells. (**E**) In the MTT assay, FXN protects against ZEA cytotoxicity (24 h). (**F**) HepG2 morphological changes. Values are presented as the mean and standard error of the mean (SD). One-way analysis of variance followed by Tukey’s post hoc test analysis was used for inter-group comparison. A significant difference is defined as a difference between the mean and standard deviation of three duplicates (*n* = 3), with * *p* < 0.05 control vs. ZEA and # *p* < 0.0.5ZEA vs. pre-treated.

HepG2 cells were found to have reduced viability when treated with ZEA. The reduction in cell viability was associated with higher concentrations of ZEA, leading to a greater decrease in cell viability. In contrast, cells treated with only FXN alone exhibited no significant difference in viability from the control cells (Figure 1D), and when treated with FXN and ZEA, FXN significantly protected cells from ZEA-induced cell death (Figure 1E). HepG2 morphology was changed by ZEA (40 µM), as shown in Figure 1F. Apoptotic cells exhibit characteristics similar to those of the membrane blebs. A normal architecture was observed in the FXN cells treated with ZEA. The effect of FXN was significantly and remarkably suppressed at a higher concentration (50 µM). It is evident from the above data that FXN might be a potent inhibitor of liver damage caused by ZEA.

### 2.2. FXNReduced ZEA-Induced Cytokine Production

**Figure 2:**
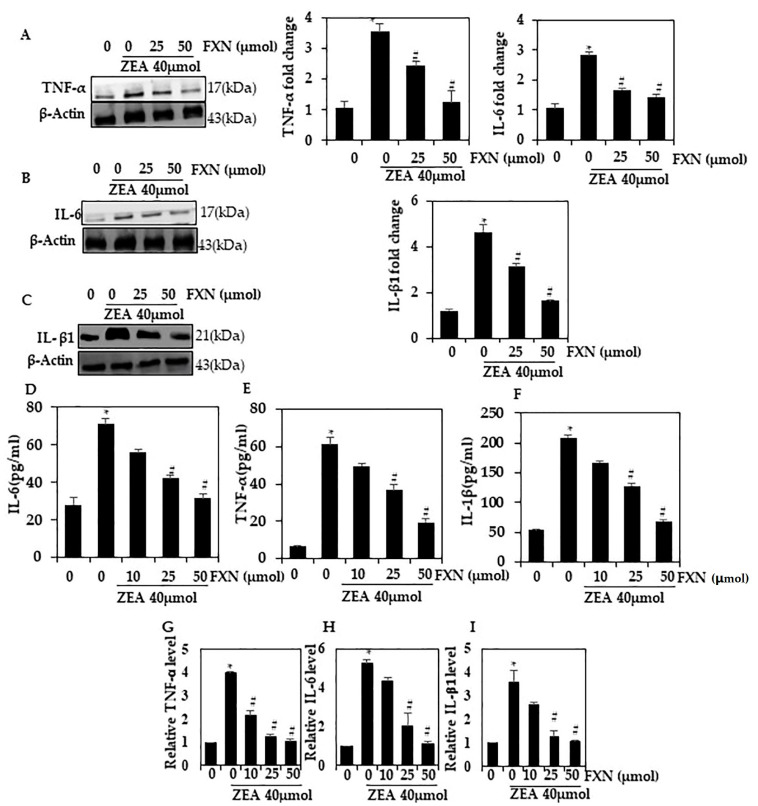
Hepatic cells were protected from ZEA-induced cytokine production by FXN. FXN and/or ZEA were administered to HepG2 cells for 24 h. Western blotting was used to assess the expression of TNF-α, IL-6, and IL-β1 proteins in (**A**–**C**). (**D**–**F**) IL-6, IL-1β, TNF-α, and VCAMsecretion into the culture media was quantified using a commercial ELISA kit. (**G**–**I**) mRNA level of the pro-inflammatory cytokines TNF-α, IL-6, and ILβ1 by RT-PCR. Values are presented as the mean and standard error of the mean (SD). One-way analysis of variance followed by Tukey’s post hoc test analysis was used for inter-group comparison. A significant difference is defined as a difference between the mean and standard deviation of three duplicates (*n* = 3), with * *p* < 0.05 control vs. ZEA and # *p* < 0.05 ZEA vs. pre-treated.

HepG2 cells were tested for inflammation induced by ZEA by Western blot. There was an increase in TNF-α, IL-1β, and IL-6 levels in cells treated with ZEA (*p* < 0.05). When HepG2 was pre-incubated with FXN, the levels of TNF-α, IL-6, and IL-1β were significantly reduced (*p* < 0.05) compared with ZEA alone (Figure 2A–C). The inhibition of cytokine production by 50 µM FXN was greater than that by 25 µM FXN. As a result of these findings, it appears that FXN reduces inflammation induced by ZEA through a reduction in cytokine expression.

### 2.3. FXN Effects on Cytokine mRNA Levels Induced by ZEA

Additionally, we investigated whether FXN inhibited the activation of the inflammatory pathway in cells containing ZEA supplements. RT-PCR analysis showed a significant reduction in TNF-α, IL-6, and IL-β1 mRNA in FXN-pre-treated cells (25 and 50 µM) (*p* < 0.05) compared to ZEA-treated cells (Figure 2D–F). A 50 µM concentration of FXN caused a greater inhibition than a 25 µM concentration. According to these data, we reconfirm FXN downregulated inflammation responses to ZEA.

### 2.4. FXN Inhibits ROS Formation in ZEA-Induced Hepatic Cells

**Figure 3:**
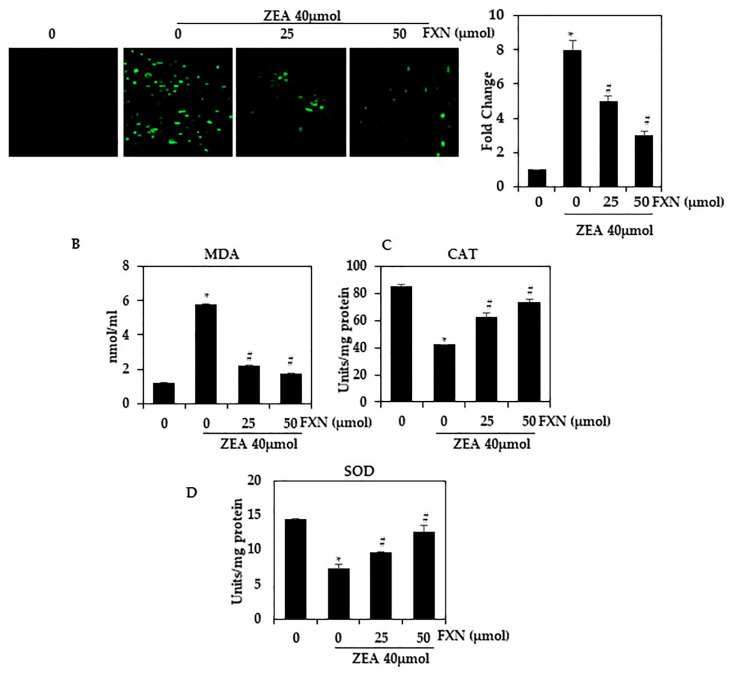
Effect of FXN on ZEA-induced ROS production in HepG2 cells. As shown in (**A**), HepG2 cells were then pre-treated with FXN (0, 25, and 50 µM) for 2 h, followed by ZEA (40 µM) for 4 h. H2DCFDA fluorescence was used to measure intracellular ROS levels. (**B**–**D**) MDA, SOD, and CAT. Values are presented as the mean and standard error of the mean (SD). One-way analysis of variance followed by Tukey’s post hoc test analysis was used for inter-group comparison. A significant difference is defined as a difference between the mean and standard deviation of three duplicates (*n* = 3), with * *p* < 0.05 control vs. ZEA and # *p* < 0.05 ZEA vs. pre-treated.

To determine the inhibitory effect of FXN on ZEA-induced oxidative stress, HepG2 cells were labeled with H2DCFDA. ROS production was visualized using fluorescence microscopy (Figure 3A). As shown in Figure 3A, ZEA significantly increased intracellular ROS levels compared with untreated cells. ZEA-induced ROS levels were significantly reduced by pretreatment with FXN (25 and 50 µM) (*p* < 0.05). Treatment of cells with 50 µM FXN caused more inhibition than treatment with 25 µM FXN.

ZEA significantly reduced SOD and CAT levels compared to the control (*p* < 0.05). A significant increase in these enzyme levels was observed in ZEA-HepG2 cells treated with FXN (*p* < 0.05) (Figure 3C–D). It has been demonstrated that FXN suppresses oxidative stress induced by ZEA in HepG2 cells. The antioxidant effect of FXN on ZEA-treated cells may be related to hepatocyte protection against oxidative stress. Through enhancing antioxidant enzymes, hepatocytes have developed a defense system against oxidative stress. This suggests that FXN may be a potential therapeutic agent for the treatment of ZEA-associated hepatic diseases.

### 2.5. Effects of FXN on NF-кB Activation in ZEA-Induced Hepatic Injury

**Figure 4:**
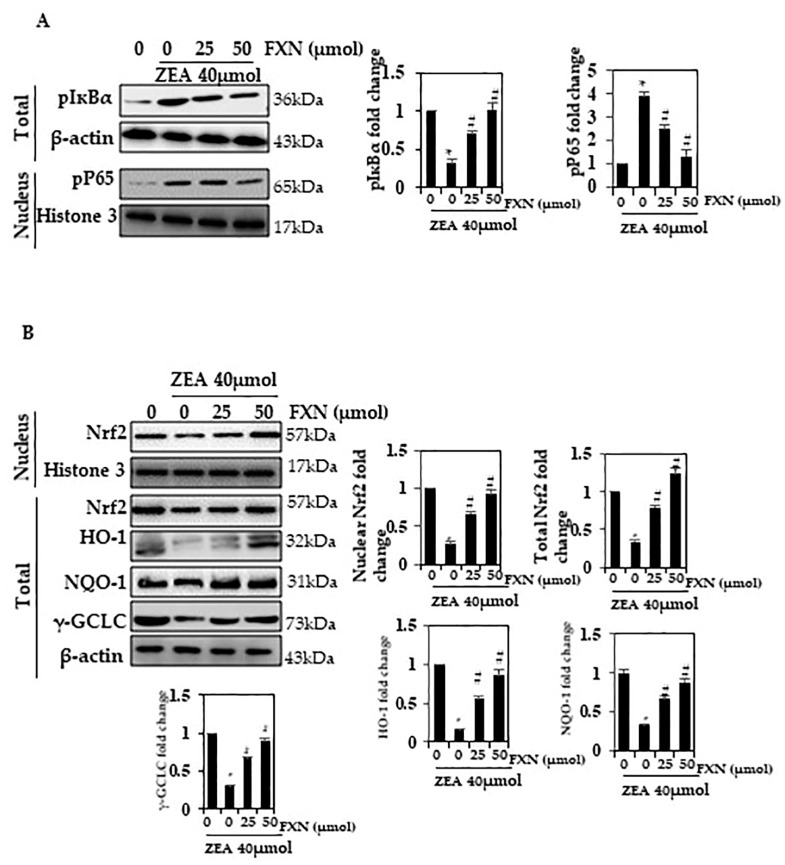
An altered FXN response to NF-кB p65 activation. (**A**) Nuclear protein extract and total protein extract were detected using p-IкBα and pNF-кB p65 antibodies on SDS-PAGE. (**B**) Western blot analysis was conducted to determine nuclear Nrf2, and total NQO-1, HO-1, and γ-GCLC. Values are presented as the mean and standard error of the mean (SD). One-way analysis of variance followed by Tukey’s post hoc test analysis was used for inter-group comparison. A significant difference is defined as a difference between the mean and standard deviation of three duplicates (*n* = 3), with * *p* < 0.05 control vs. ZEA and # *p* < 0.05 ZEA vs. pre-treated.

We investigated the role of FXN in NF-кB activation in this study. A decrease in phosphorylation of IкBα in the total extract of cells pre-treated with FXN (25 and 50 µM) was observed (Figure 4A). (*p* < 0.05). In this study, it was found that ZEA increased phosphorylation, while FXN inhibited it. There was more inhibition when cells were treated with 50 µM FXN than when they were treated with 25 µM FXN.

### 2.6. Effects of FXN on Nrf2 Activation in ZEA-Induced Hepatic Injury 

Cells require Nrf2 to function, which plays a role in oxidative stress. It has been reported that Nrf2 is capable of regulating oxidative stress by inducing the activation of a variety of protective proteins (γ-GCLC,HO-1, and NQO1) during oxidative stress. We further assessed whether FXN would have an active role in Nrf2, HO-1, and NQO1 activation. Western blotting analysis demonstrated that FXN significantly upregulated Nrf2, HO-1, γ-GCLC, and NQO1 activation, as confirmed in Figure 4B.

### 2.7. In ZEA-Induced Hepatocytes, FXN Promotes PI3K/AKT Phosphorylation

**Figure 5:**
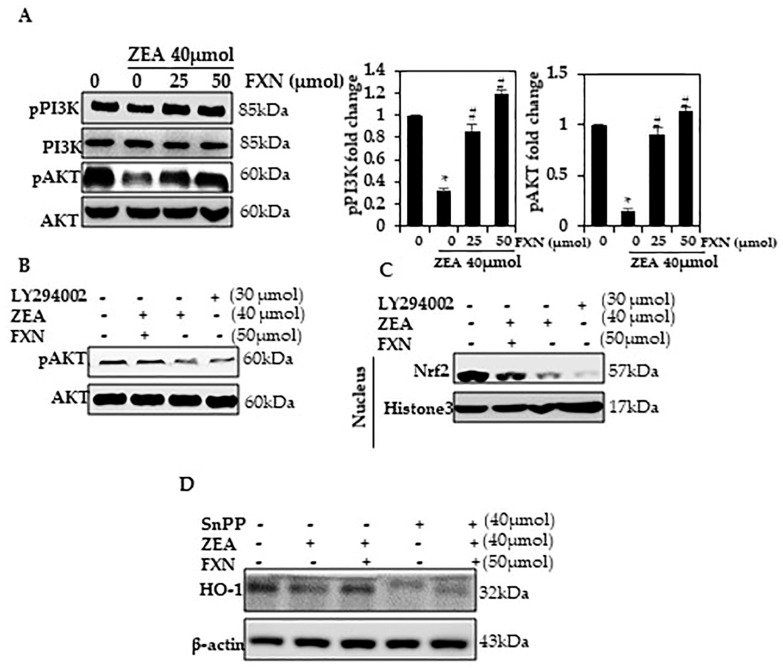
PI3K/AKT phosphorylation is activated by FXN. (**A**) The cells were treated with ZEA or FXN for 24 h. Western blotting results list the relative ratios of PI3K and AKT expression. (**B**,**C**) The cells were pre-treated with PI3K/AKT inhibitors for 2 h and then treated with FXN with or without ZEA for 24 h. Western blotting detected pAKT and Nrf2. (**D**) A Western blot was conducted after cells were exposed to SnPP for 1 h, followed by treatment with ZEA and FXN for 24 h. Values are presented as the mean and standard error of the mean (SD). One-way analysis of variance followed by Tukey’s post hoc test analysis was used for inter-group comparison. A significant difference is defined as a difference between the mean and standard deviation of three duplicates (*n* = 3), with * *p* < 0.05 control vs. ZEA and # *p* < 0.05 ZEA vs. pre-treated.

There is a lot of evidence that ROS activate apoptosis through the PI3/AKT pathway [27,28,29,30]. A number of transcription factors, including Nrf2, are inactivated by oxidative stress, including PI3/AKT phosphorylation [31,32,33,34]. As a test of whether ZEA affects PI3/AKT phosphorylation expression, HepG2 cells were induced and exposed to ZEA. PI3/AKT expression was significantly decreased after exposure (*p* < 0.05) as determined by Western blotting (Figure 5A). Upon exposure to FXN, HepG2 showed an increase in p-PI3K and p-AKT expression (*p* < 0.05). Through a signaling pathway such as PI3K/AKT, FXN appears to protect against ZEA-induced oxidative stress.

### 2.8. In ZEA-Induced Hepatocytes, FXN Activates PI3K/Akt Signaling

We determined whether Nrf2 signals are associated with the up-regulation of pAkt using Western blotting. A significant increase in pAkt, Nrf2, and HO-1 was observed among cells treated with FXN and treated with ZEA; however, LY294002 and FXN combined resulted in a significant decrease in these proteins (Figure 5B,C). In liver cells, FXN may be regulated by PI3K/Akt-Nrf2 signaling.

### 2.9. HO-1 Activation by FXN Is Regulated by the Nrf2 Pathway

Our findings led us to examine the effects of FXN on ZEA-induced oxidative stress in the presence of HO-1 inhibitors. To investigate whether FXN plays a role in ensuring HepG2 survival, we examined how Snpp affected the expression of the aforementioned protein signals. The Snpp result largely contradicts this finding (Figure 5D) as ZEA and/or FXN increased protein expression compared with FXN alone. In addition, Snpp prevented the rise in the expression of HO-1 protein caused by FXN.

## 3. Discussion

Mycotoxin toxicity has been linked to molecular mechanisms, including oxidative stress and free radical accumulation [35,36]. DNA, lipids, and proteins can be chemically impaired by free radicals and antioxidants during mycotoxin exposure [37,38,39]. Our study suggests that FXN may be a novel factor influencing hepatic tissues’ pathological condition following ZEA exposure. This study examined whether ZEA treatment significantly affected the viability of HepG2 cells. With FXN treatment, the viability was increased. This indicates that ZEA decreases the viability of hepatic cells, and that FXN may significantly reduce oxidative stress signals overproduction. According to these findings, FXN may inhibit the death of cells caused by oxidative stress induced by ZEA. In the modulation of immune responses, the cytokine family of ligands and receptors plays a pivotal role. The role of cytokine signaling in chronic liver damage and fibrosis has been demonstrated in recent research [40,41]. A number of studies have demonstrated that IL-6 and IL-1β play a crucial role in the progression of steatosis to steatohepatitis and liver fibrosis [42,43,44,45]. In addition, they play an important role in the damage caused by various toxic substances to the liver. In fibrotic livers, IL-1 induces Th2 cells and attracts innate lymphoid cells, which contribute to liver fibrosis. Rajendran et al. found that ZEA treatment increased IL-6, TNF-α, and IL-β1 levels [4]. Studies indicate that FXN has a positive effect on the reduction in inflammatory cytokines [11,15,46]. According to previous research, ZEA significantly increased TNF-, IL-6, and IL-1 expression in hepatic cells, whereas FXN treatment significantly decreased these expressions. There was an inflammatory response induced by ZEA in HepG2 cells, and the cells were protected by FXN.

At moderately low concentrations, antioxidants can compete with other oxidizable substances and reduce oxidation considerably. An antioxidant’s physiological function is to protect cellular components from ROS and free radical damage [47]. According to recent studies, oxidative stress signals, free radicals, and reactive oxygen species play an important role in the development of a number of diseases [48,49,50]. Mycotoxins are toxic and can be combated by antioxidants, most of which are natural in origin. The induction of antioxidant mechanisms by ZEA increases MDA levels in vivo and in vitro, including a reduction in CAT and SOD activity [51,52]. It has been shown that these enzymes play an important role in intracellular antioxidant activity; therefore, the augmentation of SOD and CAT functions is related to a mechanism that diminishes the accumulation of ROS that is induced by ZEA. According to our present results, ZEA causes a decrease in antioxidant levels in hepatic cells. There is a possibility that this may be due to the high levels of ROS produced by ZEA, which leads to severe glutathione depletion and facilitates the subsequent reduction in glutathione in the liver. Preventing the depletion of SOD and CAT pre-administration of FXN exerts a protective mechanism that increases antioxidant activity. It is believed that this is because FXN is capable of protecting against oxidative damage, inhibiting membrane peroxidation, and exhibiting membrane-stabilizing properties.

PI3K/AKT signaling pathways, as well as HO-1/Nrf-2 signaling pathways, may be involved in apoptosis suppression. In addition, HO-1/Nrf-2 expression and biological effects of FXN have been reported in different cell lines [53,54,55]. ZEA reduced the expression of NQO-1, HO-1, GCLC, and Nrf-2 proteins in HepG2 cells compared to cells treated with ZEA and FXN. HO-1 is the rate-limiting enzyme in heme catabolism. In addition to exerting cytoprotective, anti-inflammatory, and antioxidative properties, HO-1 can also inhibit apoptosis. Other cell lines have demonstrated a link between modulating HO-1 and the production of ROS [56,57,58,59,60]. As a result, the upregulation of HO-1 via FXN could contribute to attenuating intracellular ROS production in FXN with ZEA. As a result of FXN exposure, Nrf-2 expression significantly increased compared with ZEA incubation alone. The results of this study demonstrate that FXN inhibits oxidative stress by affecting the HO-1/Nrf-2 system.

## 4. Materials and Methods

### 4.1. Reagents

The Chinese Academy of Sciences in Shanghaiprovided HepG2.Sigma (St Louis, MO, USA) providedZearalenone (Z2125) and FXN (3351-86-8). We purchased H2DCFDA (CAS 4091-99-0) from Sigma and MTT and DMEM from Gibco BRL/Invitrogen (Waltham, MA, USA). Analytical-grade chemicals were used throughout.

### 4.2. Culture and Treatment

HepG2s were cultured at 37 °C with 10% FBS in DMEM. After 24 h, a serum-free medium was used to suspend FXN and ZEA. Cells were seeded into culture plates, and then FXN, ZEA, or a combination of the two was added over 24 h in triplicate.

### 4.3. Cell Viability Assay by Using MTT Assay

In order to determine the cytotoxic effects of FXN and ZEA on HepG2 cells, an MTT kit was used in accordance with the manufacturer’s instructions. We incubated 18-passage HepG2 cells in 96-well plates (5 × 10^3^ cells per well) for 24 h with different concentrations of ZEA and/or FXN for 24 h. Microplate readers (Winoosky, VT, USA) were used to measure the absorbance at 570 nm in each well.

### 4.4. Western Blot

Western blot analysis was performed according to the methods reported by Rajendran etal. [26]. In Buffer I (25 mM HEPES pH 7.9, 5 mM KCl, 0.5 mM MgCl_2_, and 1mm dithiothreitol (DTT)), cytoplasmic material was extracted for 5 min. Afterwards, it was mixed with an equivalent volume of Buffer II (25 mM HEPES pH 7.9, 5 mM KCl, 0.5 mM MgCl_2_, 1 mM DTT, and 0.4% (*v*/*v*) NP-40) with protease and phosphatase inhibitors, and kept at 4 °C under continuous stirring. We centrifuged the cell lysates for 5 min at 4 °C and 500× *g*. Subsequently, fresh 1 mL Eppendorf tubes were used to collect the supernatants. The lower layer (cell pellets) was suspended in fresh Buffer II solutions and then mixed with the cytoplasmic protein. Subsequent centrifugation at 10,000 g was performed in order to eliminate any residual nuclei from the pellets. A cytoplasmic extraction pellet was re-suspended in Buffer III (25 mM HEPES pH 7.9, 400 mM NaCl, sucrose (10%), NP-40 (0.05%), and 1 mM DTT) with inhibitors of both phosphate and protease. After vortexing for 1 h at 4 °C, the tubes were subjected to a 10min centrifugation step at 10,000 g. As a result of this process, supernatants containing nuclear proteins, nuclear Nrf2 and p-P65, were also analyzed through Western blotting. Following the treatment, cells were harvested, washed once with cold PBS, and then cytoplasmic, nuclear, and total extracts were prepared as described.

The Bio-Rad protein assay was used to detect the protein level in each sample, and bovine serum albumin was used as a reference standard. We resolved equal amounts of protein (40 µg) via SDS-PAGE (8–15%) and transferred the proteins to nitrocellulose membranes overnight. The primary antibodies TNF-α, IL-6, IL-1β, HO-1, NQO-1, γ-GCLC, p-P65, p-IKBα, pPI3K, PI3K, pAKT, and AKt were purchased from Thermo Fisher Scientific, Inc. (Waltham, MA, USA) (catalogue number: #PA5-19810, #P620, #PBOIL1BI, PA5-77833, #PA5-82294, #PA5-44190, #MA5-15160, #PA5-36653, PA5-104853, #PA5-104853, #PA5-29220, Cat #44-602G, and Cat #44-609G, respectively). Nrf2 and β-actin were purchased from MOLEQULE-ON (New Lynn, Auckland, New Zealand). After blocking the membranes at 37 °C for 30 min, the membranes were incubated with the indicated primary antibodies for 24 h. An enhanced chemiluminescence substrate (Pierce Biotechnology, Rockford, IL, USA) was used to develop the membranes after incubation with goat anti-mouse or rabbit secondary antibodies for 1 h. The samples were examined using anLI-COR chemiluminescence imaging system (3600-00-C-Digit Blot Scanner, LI-COR Biosciences, Lincoln, NE, USA). For the generation of the graphs of the densitometric band intensities, Image Studio Lite software (LI-COR Biosciences, Lincoln, NE, USA) was used. 

### 4.5. Detection and Measurement of ROS Generation

DCFH2-DA fluorescence dye method measured intracellular ROS accumulation [61]. Cells were seeded in a 6-well plate at 1 × 10^5^ cells/mL, pretreated with different concentrations of FXN (for 2 h), and then treated with or without ZEA. The incubation was completed at 37 °C for 30 min after DCFH2-DA was added to the culture medium. Using a fluorescence microscope ((200× magnification), Olympus, Center Valley, PA, USA), the dichlorofluorescein (DCF) fluorescence intensity was measured in HepG2 cells.

### 4.6. RT-PCR

For 24 h, HepG2s cells were treated with ZEA and/or FXN. As part of the reverse chain reaction, Taq polymerase and superscript reverse transcriptase were used to convert RNA into cDNA. Analysis of the relative expression of TNF-, IL-6, and IL-1 was conducted using RT-PCR. RT-PCR analysis was performed according to the methods reported by Wang et al. [62]. GAPDH was used as the housekeeping gene for the measurement of mRNA expression. Using the two-tailed Ct values, we compared the expression of mRNA between groups.

### 4.7. Statistics

Values are presented as the mean and standard error of the mean (SD). One-way analysis of variance followed by Tukey’s post hoc test analysis was used for inter-group comparison. A significant difference is defined as a difference between the mean and standard deviation of three duplicates (*n* = 3), with * *p* < 0.05 control vs. ZEA and # *p* < 0.05 ZEA vs. pre-treated.

## 5. Conclusions

FXN has been shown to be an effective additive in removing ZEA contamination in feeds, as well as protecting against ZEA toxic effects in vitro. FXN was found to be protective against oxidative hepatitis caused by ZEA through its antioxidant properties. PI3K/Akt-mediated Nrf2 activation by FXN alleviates ZEA-induced liver alterations. ROS-mediated inflammation was inhibited by FXN through Nrf2 activation, which inhibited ROS-mediated inflammation. In the future, further research may establish bioactive FXN as a possible therapy for ZEA-induced oxidative stress and hepatic complications. 

## Data Availability

Not applicable.
